# The efficacy and safety of Chinese herbal medicine for reducing wound complications after anal fistula surgery: A protocol for systematic review and meta-analysis

**DOI:** 10.1097/MD.0000000000032021

**Published:** 2022-12-02

**Authors:** Wenyue Qi, Linyue Wang, Jiancheng Xu, Jihua Gao

**Affiliations:** a Hebei University of Chinese Medicine, Hebei, China; b Department of Proctology, the First Affiliated Hospital of Hebei University of Chinese Medicine, Hebei, China.

**Keywords:** anal fistula, Chinese herbal medicine, meta-analysis, wound

## Abstract

Anal fistula is one of the most common diseases in anorectal surgery. The wound healing after surgery can affect the prognosis of patients. We conducted a protocol for systematic review and meta-analysis to assess the efficacy and safety of Chinese herbal medicine for reducing wound complications after anal fistula surgery.

**Methods:**

We have prepared this protocol in accordance with the Preferred Reporting Item for Systematic Review and Meta-analysis (PRISMA-P) statement. We will search the following databases: the China National Knowledge Infrastructure, Wanfang Database, Chinese Science and Technology Periodical Database, Chinese Biomedical Literature Database, Pubmed, Embase, Web of Science, and the Cochrane library. Two authors will independently assess the risk of bias of the included studies based on the bias risk assessment tool recommended in the Cochrane “Risk of bias” assessment tool. All calculations are carried out with STATA13.0 software.

**Results:**

A synthesis of current evidence of Chinese herbal medicine for wound management after anal fistula surgery will be shown in this protocol.

**Conclusion:**

This study may provide more convincing evidence to help clinicians make decisions when dealing with anal fistula patients after surgery.

## 1. Introduction

Anal fistula is one of the most common diseases in anorectal surgery.^[1]^ Delayed healing and other complications may occur in some patients, reducing the quality of life.^[2,3]^ The healing status after anal fistula surgery is related to many factors, and the surgical method is only 1 aspect that affects the healing rate and recurrence rate after anal fistula surgery.^[4]^ The nutritional status of patients and the local microenvironment of wounds are also important factors affecting healing, such as systemic hyperglycemia in diabetes patients and local inflammation of Crohn’s anal fistula.^[5]^ The immuno-inflammatory response is a prerequisite for wound healing and tissue regeneration. Systemic and topical immune regulation could improve the health status of patients and the healing environment of local wounds.^[6,7]^ Lymphocyte count is an effective predictor of body nutrition, immune status, and inflammatory response. Patients with a reduced systemic lymphocyte count often suffer from immune disorders whose healing ability of incisions or wounds is decreased.^[8]^ However, lymphocyte aggregation and the change of subtype ratio in the local wound are also important manifestations of the inflammatory response, which can often delay healing. Changes in systemic lymphocytes and their subsets have important significance in predicting systemic infection and the risk of related death. However, the association between local lymphocyte count in the wound and the healing time of the wound after anal fistulotomy remains unclear.

Traditional Chinese medicine (TCM) has a long history in the treatment of anal fistula, which can not only relieve postoperative pain but also significantly shorten the course of the disease.^[9,10]^ TCM has attracted the most attention for western countries in these years because of its reliable therapeutic efficacy and fewer side effects. In this study, we conducted a protocol for systematic review and meta-analysis to assess the efficacy and safety of Chinese herbal medicine for reducing wound complications after anal fistula surgery.

## 2. Methods

### 2.1. Study registration

This systematic review was registered on PROSPERO (CRD42022371866) in October 2022. We have prepared this protocol in accordance with the Preferred Reporting Item for Systematic Review and Meta-analysis (PRISMA-P) statement,^[11]^ and we will update the PROSPERO record if there are any important amendments. Ethical approval is not required for this study since it relies on secondary data.

### 2.2. Inclusion criteria

Type of studies: randomized trials and quasi-randomized or prospective controlled clinical trials that have tested traditional Chinese herbal medicine with or without western medicine for anal fistula after surgery will be included. There will be no restrictions for blinding, follow-up, or publication status. Publications in English and Chinese will be included. Type of participant: patients diagnosed with anal fistula undergoing surgery will be included. There will be no restrictions with respect to gender, age, or ethnicity. Type of interventions: patients in experimental group received traditional Chinese herbal medicine involving extracts from herbs, single or mixture herbal formulas regardless of their compositions or forms. Traditional Chinese herbal medicine combined with one or more other pharmacological intervention will also be included. There will be no restrictions with respect to dosage, frequency, duration, or follow-up time of treatment. Type of comparators: there will be no restrictions with respect to the type of comparator. The comparators are likely to include western medical therapies, supportive care, and other therapeutic methods. Type of outcome measurements: the mains outcomes include pain score, wound complication such as edema, and exudation. The additional outcomes include healing time of surgical wound, function of anal sored and quality of life.

### 2.3. Data sources and search strategies

We will search the following databases: the China National Knowledge Infrastructure, Wanfang Database, Chinese Science and Technology Periodical Database, Chinese Biomedical Literature Database, Pubmed, Embase, Web of Science, and the Cochrane library. The retrieval time is from the inception of the database to October, 2022. The language is limited to Chinese and English. Reference lists of relevant trials and reviews will be searched. The search strategy is to combine search terms with subject words and free words. The primary selection process are shown in PubMed search strategy (Table 1).

**Table 1:** Search strategy for the PubMed database.

#1 traditional Chinese medicine [Title/Abstract]
#2 Chinese herb medicine [Title/Abstract]
#3 TCM [Title/Abstract]
#4 #1 OR #2 OR #3
#5 anal fistula [Title/Abstract]
#6 fistula-in-ano [Title/Abstract]
#7 anorectal fistula [Title/Abstract]
#8 ligation therapy [Title/Abstract]
#9 anal fistulectomy [Title/Abstract]
#10 #5 OR #6 OR #7 OR#8 OR#9
#11 wound [Title/Abstract]
#12 healing [Title/Abstract]
#13 complication [Title/Abstract]
#14 infection [Title/Abstract]
#15 #11 OR #12 OR #13 OR #14
#16 randomized controlled trial[Publication Type]
#17 randomized [Title/Abstract]
#18 randomly [Title/Abstract]
#19 #16 OR #17 OR #18
#20 #4 AND #10 AND #15 AND #19

### 2.4. Study selection

**Figure 1.**
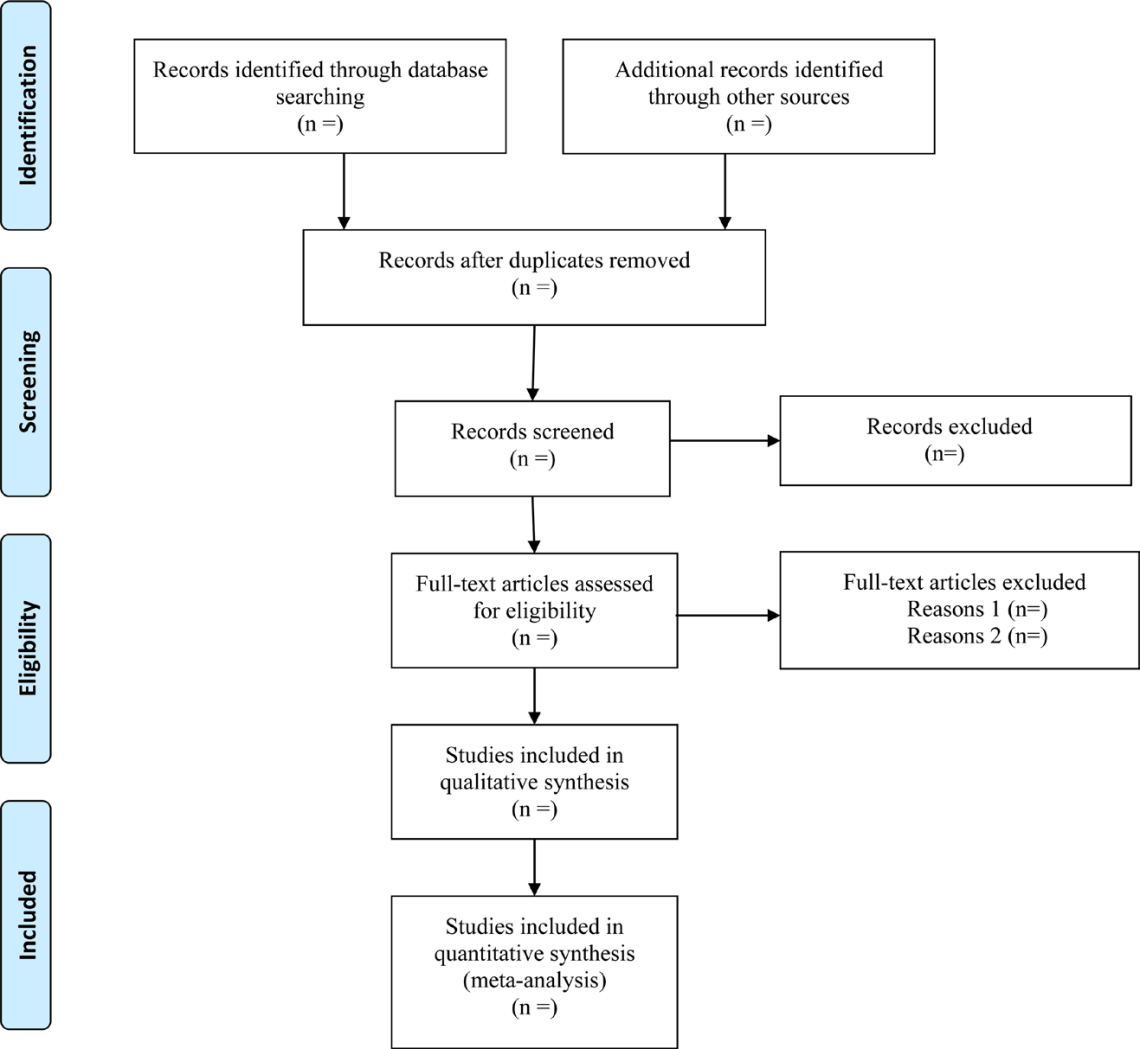
PRISMA flow chart of study selection process. PRISMA = preferred reporting item for systematic review and meta-analysis.

We will export the identified records in databases into EndNote X9 software and use this to identify duplicates. After removing duplicates, the retrieved records will be checked independently by 2 reviewers, who will apply the eligibility criteria based on the title and abstract. Where a study is potentially eligible, the full text will be obtained and checked independently by 2 reviewers to identify the eligible studies. Any disagreements will be discussed and resolved in discussion with a third reviewer. Details of the selection procedure for the studies are shown in the PRISMA flow chart (Fig. 1).

### 2.5. Data extraction

In order to achieve a consistency of extracted items, the data extractors will extract data from a sample of eligible studies. Results of the pilot extraction will be discussed among review authors and extractors. Two independent reviewers will extract data with a predefined extraction template, which includes the following items: general information—first author, title, journal, year of publication, country, funding source, study design, etc; characteristics of patients—age, gender, race and severity of disease; characteristics of intervention—protocol of Chinese herbal medicine (types, dosage, frequency, duration, etc) and protocol of comparators (types, dosage, frequency, duration, etc); characteristics of trial—sample size, generation of randomization sequence, allocation concealment, blinding, duration of follow-up, etc; and outcomes—all outcomes, main conclusions, adverse events, etc. The original authors will be contacted to request missing data where necessary. Any disagreements will be discussed and resolved in discussion with a third reviewer.

### 2.6. Risk of bias assessment

Two authors will independently assess the risk of bias of the included studies based on the bias risk assessment tool recommended in the Cochrane “Risk of bias” assessment tool.^[12]^ Including 7 items: random sequence generation, allocation concealment, blind participants and personnel, blind assessment of results, incomplete result data, selective reports, and other biases. The results in each field will be divided into 3 levels: low bias risk, high bias risk, and unclear bias risk.

### 2.7. Statistical analysis

Two researchers respectively entered the data into the STATA13.0 software. Mean differences (MDs) with a 95% confidence interval (CI) were calculated to assess the effect size for continuous outcome data. Risk ratio with a 95% CI were used as effect size for dichotomous data. Inverse variance method and Mantel-Haenszel analysis method were used for continuous variables and dichotomous variables, respectively.^[13]^ The heterogeneity among the trials was assessed for significance with *Q* and quantified with *I*^2^. Statistically significant was set at the *P* value <.10. If the studies were homogeneous or the statistical heterogeneity was low, we used the fixed effect-model. While, random-effects model was applied when the statistical heterogeneity was moderate or high.

### 2.8. Subgroup analysis

If the heterogeneity of the included studies is large, subgroup analyses will be performed on the basis of different interventions, controls, durations of treatment, and outcome measures.

## 3. Discussion

Anal fistula is one of the most common diseases in young adults. Anal fistula cannot heal itself without treatment and will recur; therefore, radical surgery should be performed promptly.^[14,15]^ The wound surface after anal fistula surgery is usually open which makes it susceptible to bacterial attack from feces and intestinal bacteria.^[16,17]^ This prolongs the wound healing time, which is compounded by abundant perianal nerve endings which makes the postoperative pain intense. Therefore, the wound healing after anal fistula surgery can affect the prognosis of patients. Similar to other surgical wounds, wound healing after anal fistula surgery can be divided into 3 stages, that is, inflammatory exudate, fibrous tissue hyperplasia, and scar formation.^[18,19]^ Targeted intervention can effectively relieve postoperative pain, accelerate the disappearance of symptoms, relieve the pain of patients, and promote rapid wound healing.

To the best of our knowledge, this is the first meta-analysis to assess the efficacy and safety of Chinese herbal medicine for reducing wound complications after anal fistula surgery. We hope that it will provide more convincing evidence to help clinicians make decisions when dealing with anal fistula patients after surgery.

## Author contributions

**Conceptualization:** Linyue Wang.

**Formal analysis:** Jiancheng Xu.

**Writing – original draft:** Wenyue Qi.

**Writing – review & editing:** Jihua Gao.
